# Next-generation 2D optical strain mapping with strain-sensing smart skin compared to digital image correlation

**DOI:** 10.1038/s41598-022-15332-1

**Published:** 2022-07-03

**Authors:** Wei Meng, Ashish Pal, Sergei M. Bachilo, R. Bruce Weisman, Satish Nagarajaiah

**Affiliations:** 1 Department of Civil and Environmental Engineering, Rice University, Houston, TX 77005 USA; 2 Department of Chemistry, Rice University, Houston, TX 77005 USA; 3 Department of Materials Science and NanoEngineering, Rice University, Houston, TX 77005 USA; 4 Department of Mechanical Engineering, Rice University, Houston, TX 77005 USA

**Keywords:** Aerospace engineering, Civil engineering, Mechanical engineering

## Abstract

This study reports next generation optical strain measurement with “strain-sensing smart skin” (S^4^) and a comparison of its performance against the established digital image correlation (DIC) method. S^4^ measures strain-induced shifts in the emission wavelengths of single-wall carbon nanotubes embedded in a thin film on the specimen. The new S^4^ film improves spectral uniformity of the nanotube sensors, avoids the need for annealing at elevated temperatures, and allows for parallel DIC measurements. Noncontact strain maps measured with the S^4^ films and point-wise scanning were directly compared to those from DIC on acrylic, concrete, and aluminum test specimens, including one with subsurface damage. Strain features were more clearly revealed with S^4^ than with DIC. Finite element method simulations also showed closer agreement with S^4^ than with DIC results. These findings highlight the potential of S^4^ strain measurement technology as a promising alternative or complement to existing technologies, especially when accumulated strains must be detected in structures that are not under constant observation.

## Introduction

A stress concentration is a location at which the mechanical stress is significantly higher than in the surrounding area. It can occur when there are irregularities in the geometry or material of a structural component. Brittle materials will typically fail at such high stress locations due to fracture and cracking. For ductile materials, stress concentration might instead cause localized plastic deformation and yielding. Moreover, fatigue and fracture cracks due to low level but high-frequency loads would also grow in stress concentration regions and cause damage. Many cases of structural failure in buildings, bridges, ships, and aircraft are closely related to stress–strain concentration. As a direct indicator of the effects of stress concentration, strain measurement plays an important role in structural health monitoring (SHM) and non-destructive testing. For this reason, many analytical, numerical^1–3^, and experimental studies have been conducted over the past decades to investigate structural strain and damage induced by various loading conditions.

Experimental strain measurement methods can be divided into two major categories: contact-based and non-contact techniques. In contact-based strain sensing, piezoresistive and fiber Bragg grating (FBG) sensors are the most widely used. Piezoresistive strain sensors include the foil strain gauge and other sensors fabricated from materials with piezoresistive properties, such as carbon nanotubes (CNTs)^4–6^ and metal compounds ^7–9^. In piezoresistive materials, the conductivity changes with strain in a linear relationship^10^. By contrast, FBG sensors are optical, offering the advantages of insensitivity to electromagnetic interference, small dimensions, and resistance to corrosion^11–13^. However, for both piezoresistive and FBG sensors, the strain is measured pointwise in an individual direction, which leads to high cost and low spatial resolution when full-field strain mapping is needed. These sensors are most useful when users have prior knowledge of the locations of stress concentration and can deploy them accordingly.

Optical non-contact strain sensing techniques have two main advantages. One is avoiding the need for electrical or fiber optic connections between the sensors and the measuring device. The other is showing the strain distribution over a two-dimensional region of interest, which is important for damage detection and studies of fracture behavior. Currently, the full-field optical non-contact strain sensing techniques can be classified as: (1) interferometric^14–23^, (2) image-based, or (3) spectroscopic. Interferometric techniques measure the micrometer scale displacements of a material based on optical interference patterns. They can be highly sensitive to strain field variation, but are only suitable for small-scale model structure measurement in laboratory environments.

The most widely accepted and commonly used image-based method is digital image correlation (DIC), which provides full-field displacement and strain maps by comparing digital images of natural or applied patterns on the specimen surface in undeformed and deformed states^24^. Compared to interferometric techniques, DIC can be used in a wider range of measurement environments and for different spatial scales, strain sensitivities, and spatial resolutions. However, DIC is an indirect method that relies on complex numerical image processing. The virtual strain gauge (VSG) is a common and key element in the DIC method. VSG is a small region of the image over which average strain is calculated, analogous to the physical area covered by a resistive strain gauge. The choice of VSG size therefore determines the spatial resolution and accuracy in a DIC strain measurement. A small size generates strain maps with less smoothing at the cost of noisier strain data, while a large VSG size reduces noise but may fail to detect sharp spatial strain gradients indicative of structural damage. In practical applications, a sensitivity study on the VSG size is therefore always important for interpreting DIC results. This is usually done by comparing the results with readings from attached strain gauges used as references. Moreover, the accuracy of DIC results depends strongly on the quality of the imaging optics and camera.

In a more direct optical approach to strain measurement, spectroscopy-based methods have been developed to overcome some limitations of image-based and interferometric techniques. The leading work in this area involves single-walled carbon nanotubes (SWCNTs), which show systematic shifts in their vibrational and electronic spectra in response to mechanical deformation. SWCNTs that are attached to a surface can therefore be used as tiny, optically interrogated strain sensors. Several studies have demonstrated SWCNT-based strain sensing using shifts in the nanotubes’ vibrational Raman scattering frequencies^25–28^ in comparison with contact based approach. However, such Raman methods are hampered by intrinsically weak scattering signals and long measurement times, making them impractical for most applications. Much stronger optical signals, faster data acquisition, and higher strain sensitivity have been obtained by using SWCNT near-IR fluorescence spectra to deduce strain^29^. In this “strain-sensing smart skin” or S^4^ method, emission from SWCNTs embedded in a thin polymer film on the specimen surface is captured and spectrally analyzed to find the local strain magnitude at the desired locations and directions. Because the nanotube sensors are distributed across the entire coated surface, strain values can be measured at arbitrary locations and directions, and combined to give full-field strain maps^30–33^. We report here the latest developments in the S^4^ method and a detailed comparison of S^4^ strain mapping with results from DIC.

## New strain-sensing smart skin (S^4^) formulation

The strain sensors in our S^4^ method are single-wall carbon nanotubes. These are artificial nanomaterials with tubular structures formed from carbon atoms that are covalently bonded into specific forms with long-range crystalline order^34^. Each structural form, or species, has a distinct electronic structure and is labeled by a pair of integers, (*n*,*m*). Most SWCNT species are semiconducting and emit near-infrared fluorescence at well-defined wavelengths corresponding to their specific band baps^35^. The systematic changes in these emission wavelengths induced by SWCNT axial strain^36^ form the basis for S^4^ optical strain sensing.

**Figure 1:**
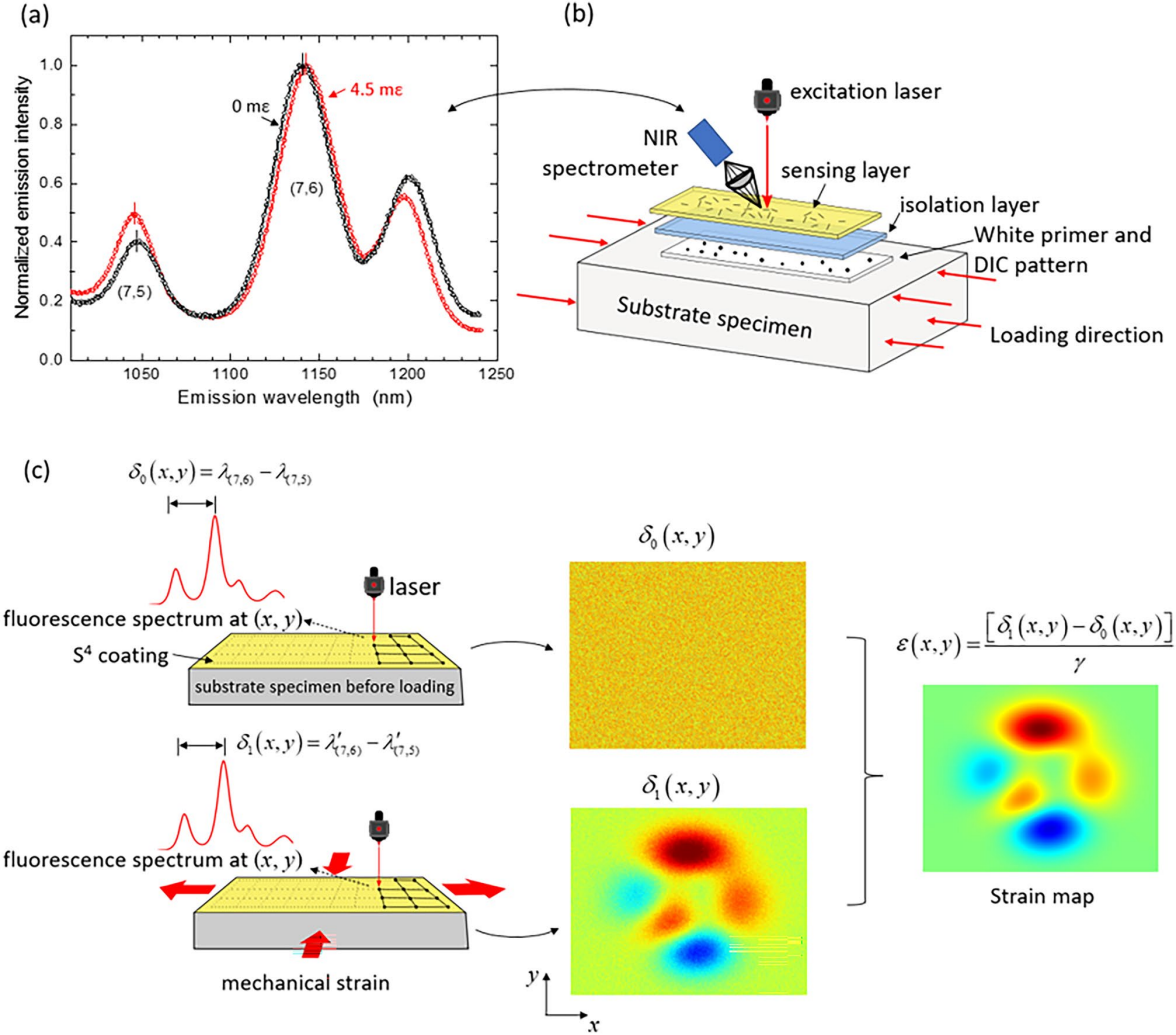
(**a**) Emission spectrum from an S^4^ film on a specimen with substrate strain of 0 (black points) and 4.5 mε (red points) [adapted from reference^30^]; (**b**) layer-structured S^4^ film; (**c**) scheme for measuring 2D strain maps in the S^4^ method. The specimen is raster-scanned before (left top) and after (left bottom) stress testing to find the spectral peak separation at each grid point. Those separations are then pointwise subtracted and divided by the spectral gauge factor to obtain the specimen’s strain map (right).

In the S^4^ films, nanotubes are individually coated with the organic polymer PFO (poly(9, 9-di-*n*-octylfluorenyl-2,7-diyl)) and applied as a toluene suspension to the specimen surface. Evaporation of the solvent leaves a submicron-thick sensing layer. Subsequent deformation of the specimen results in load transfer through the adhering polymer to the embedded SWCNTs, transmitting strain that is revealed by non-contact spectroscopic measurements of SWCNT fluorescence shifts. We have found that the wavelength separation between the (7,6) and (7,5) emission peaks, illustrated in Fig. 1a, is a reliable monitor of strain. Using the standard pre-determined spectral gauge factor that relates these peak shifts to strains, we can measure the strain magnitude and principal axis at any location on the specimen surface by positioning the fluorescence excitation beam and orienting its polarization plane.

Some substrates may be damaged by the toluene solvent or may generate luminescence that interferes with the S^4^ measurement. In such cases it is necessary to apply a base coating, such as a layer of opaque paint, to block the substrate emission and/or protect the surface. Onto this blocking base layer we apply an isolation primer layer of high gloss polyurethane to protect the blocking paint from toluene damage and to provide a microscopically smooth base for the very thin sensing layer, which is sprayed onto it after the isolation primer dries. This three-layer structure provides a consistent environment for the SWCNTs, thereby reducing variations in their initial strain states and giving good spectral uniformity without the need for annealing the film at elevated temperatures. The three-layer coating scheme also lets us paint a speckle pattern onto the blocking layer to allow parallel measurements of DIC and S^4^ strain maps on the same specimen. Figure 1b illustrates the three-layer coating structure.

To fabricate the coating, the specimen surface is first cleaned to ensure good bonding and load transfer. After that, the base coating layer is applied by spray-painting. If it is necessary to block intrinsic emission from the substrate, black paint is appropriate; otherwise, white paint is preferred to enhance the intensity of fluorescence from SWCNTs in the film. The color of speckle patterns for DIC is chosen to contrast with the base color. The speckles and primer must give matte finishes to avoid specular reflections. Other fabrication requirements such as size, density, variation, and thickness can follow the practical guidance for DIC^37^. Then the polyurethane isolation layer is sprayed over the DIC patterns. We have found good results using Minwax fast-drying clear gloss aerosol polyurethane. This layer should be approximately 2 μm thick, which is enough to smooth surface irregularities but thin enough for efficient load transfer. After the polyurethane is fully cured, which takes about 24 h at room temperature, the SWCNT sensing layer is applied using the protocol described previously^32^.

Before using the new S^4^ coating for strain measurements, we performed a simple calibration to correlate the spectral peak shifts with readings from a conventional resistive foil gauge. The change in separation between the (7,6) and (7,5) peak wavelengths is proportional to the specimen strain, with a “spectral gauge factor” defined by the slope:

1 $$ \gamma = - \frac{{d\left( {\lambda_{(7,6)} - \lambda_{(7,5)} } \right)}}{d\varepsilon } $$

Here $\gamma$_(7,6)_ and $\gamma$_(7,5)_ are the peak wavelengths of emission from (7,6) and (7,5) SWCNTs in the sensing film and *ε* is the specimen strain. The value of $\gamma$ should be consistent for different specimens prepared with the same base coating material and film application protocol.

Figure 1c illustrates the procedure used to measure strain maps using S^4^. First, before loading, a reference strain map of the specimen is obtained by point-wise raster scanning of the optical strain reader over the region of interest (ROI). At each point, the peak wavelengths of (7,6) and (7,5) SWCNT emission are determined and the wavelength difference is recorded as an element of a reference spectral array. After the specimen has been stress tested, it is scanned again with the same raster pattern to generate the final spectral array. We then subtract the reference array elements from the final array elements and divide by *γ* to obtain the array of net induced strain values. To allow comparison with a DIC strain map, we take high resolution photographs of the speckle pattern in the specimen’s ROI before and after deformation. For this, the camera position relative to the specimen needs to be precisely controlled and reproduced for the two photographs.

## Calibration procedure

**Figure 2:**
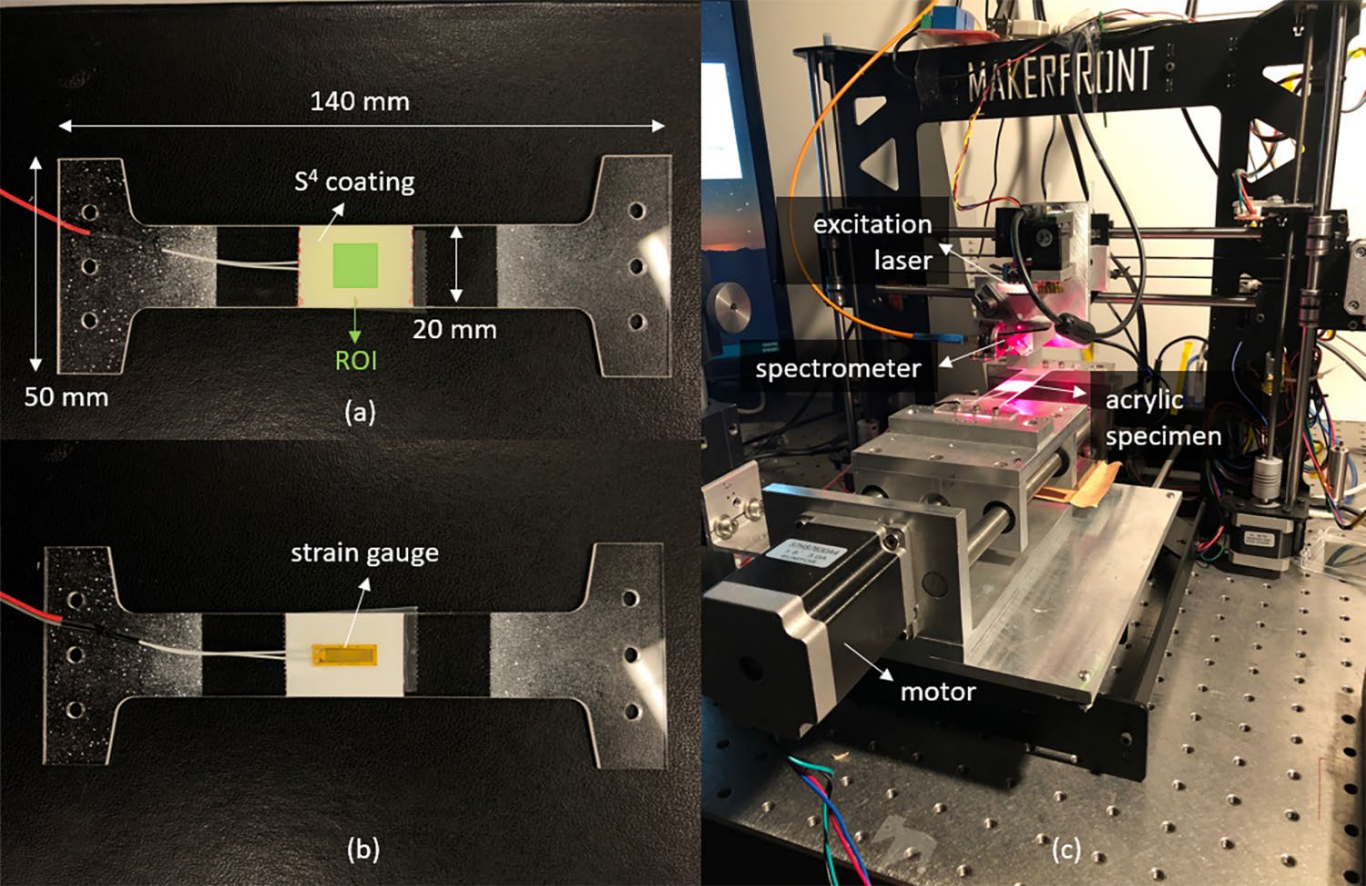
Photographs of (**a**) front face of the acrylic specimen with S^4^ coating; (**b**) back face of the acrylic specimen with attached strain gauge; (**c**) experimental setup for specimen loading and S^4^ data acquisition.

The test specimen used for calibration was a 1.4 mm thick ‘I’-shaped acrylic bar. We applied the three-layer S^4^ coating to the center of its top surface and mounted a conventional foil strain gauge to the center of its bottom surface. Figure 2 shows the specimen and the experimental setup. The bar was axially stressed to induce tensile strains from 0 to 1500 με using a motorized jig. Because the specimen did not undergo out-of-plane deformation or bending during this testing, we assume that strains were equal on its top and bottom surfaces. To assess the stability of our S^4^ measurements, we loaded and unloaded the specimen through multiple cycles while comparing S^4^ spectral data with the strains reported by the foil gauge.

The scanned ROI on this specimen was a 10 × 10 mm^2^ square area, as marked in Fig. 2. We initially adjusted the vertical read head position to give the best laser focus at the surface and then performed a horizontal raster-scan with steps of 0.5 mm, giving a total of 441 data points on a 21 × 21 point grid in the ROI. The dwell time for spectral measurement at each point was 1 s. For each measured spectrum, a custom data analysis program fit the emission features from (7,5) and (7,6) SWCNTs to Gaussian functions and precisely determined the wavelength difference between their two centers while the next spectrum was being acquired. We averaged those values over all 441 points to obtain the mean peak separation for each of several loading levels reported by the foil gauge.

**Figure 3:**
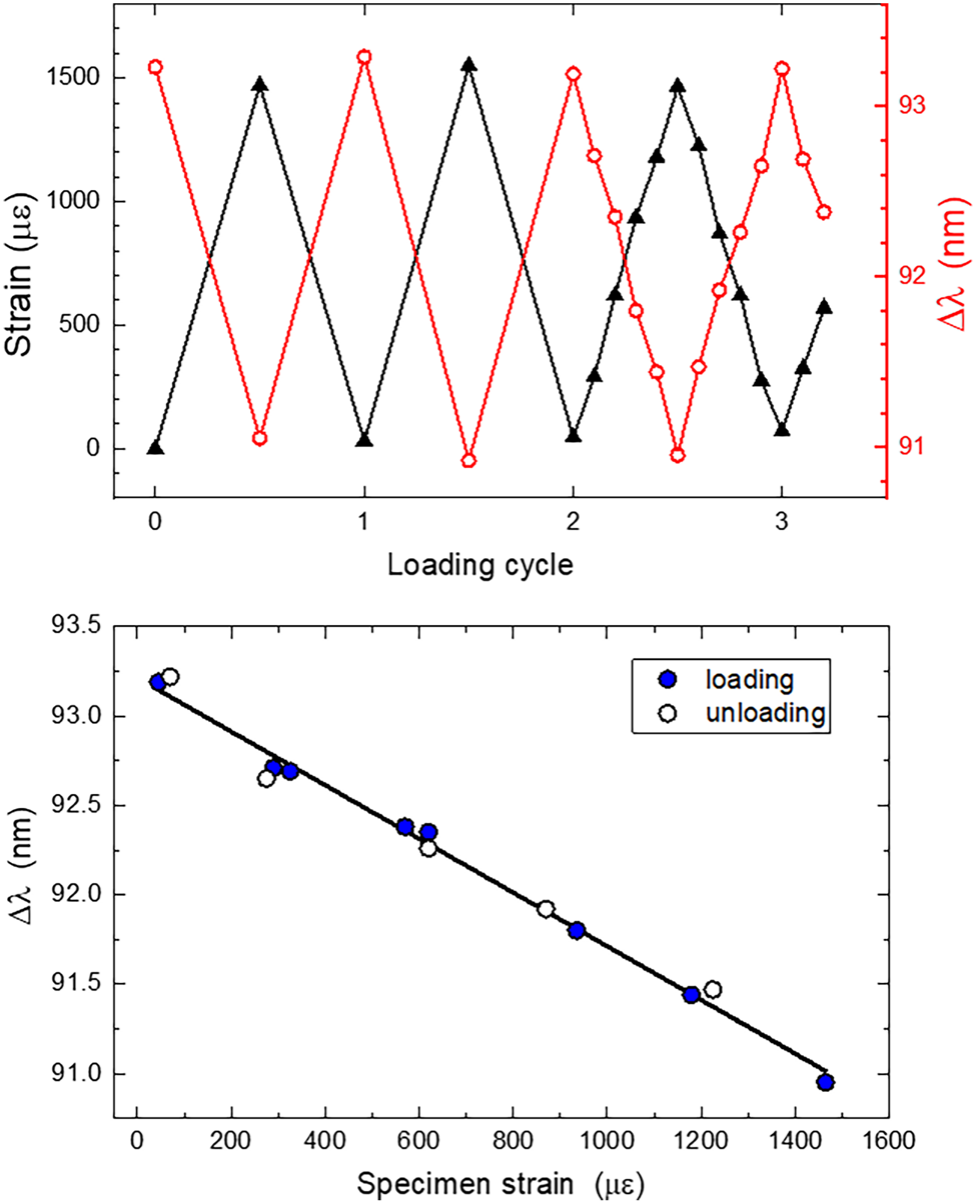
Top frame: recorded strain gauge readings (red) and averaged peak wavelength separations (black) under cyclic tensile loading; Bottom frame: peak wavelength separations vs. specimen strain for the final 1½ loading cycles (slope is − 1.5 nm/mε).

The top frame of Fig. 3 shows the foil strain gauge readings with the corresponding wavelength separations between (7,6) and (7,5) peaks measured during the cyclic loading. The wavelength separations for cycles 2 and above are plotted as a function of strain in the bottom frame. These data show a nearly linear response with a *γ* value (slope) of − 1.5 nm/mε, which is very close to the value found in prior S^4^ studies with no base layer beneath the sensing layer^32^. We found a *γ* value (slope) of − 1.5 nm/mε as “standard gauge factor” in all cases in this paper. These results also indicate efficient and reproducible load transfer from the test specimen to the nanotube strain sensors.

## Full-field strain mapping comparisons between S^4^ and DIC

We performed the following three case studies to compare strain maps measured using scanned S^4^ and DIC methods. The specimens represent different materials with shapes designed to generate distinct patterns of stress/strain concentration during testing.

### Case 1: Acrylic bar

The first comparison specimens were two ‘I’-shaped acrylic bars with the same dimensions as described above. For the first specimen, a square notch was cut into the side to concentrate stress under axial loading and thereby generate a characteristic strain pattern. For the second specimen, a circular hole was drilled at the center. The central section of the specimen’s top surface was again coated by an S^4^ film with a DIC speckle pattern applied to the base layer. We attached a conventional foil strain gauge to the bottom surface near the structural irregularities. The size of the square notch, circular hole, and position of strain gauge are shown in Fig. S1. The yielding strain of the acrylic specimen was found to be near 20 mε^38^. To keep the deformation test well within the elastic range, we limited strain to 1.7 mε.

**Figure 4:**
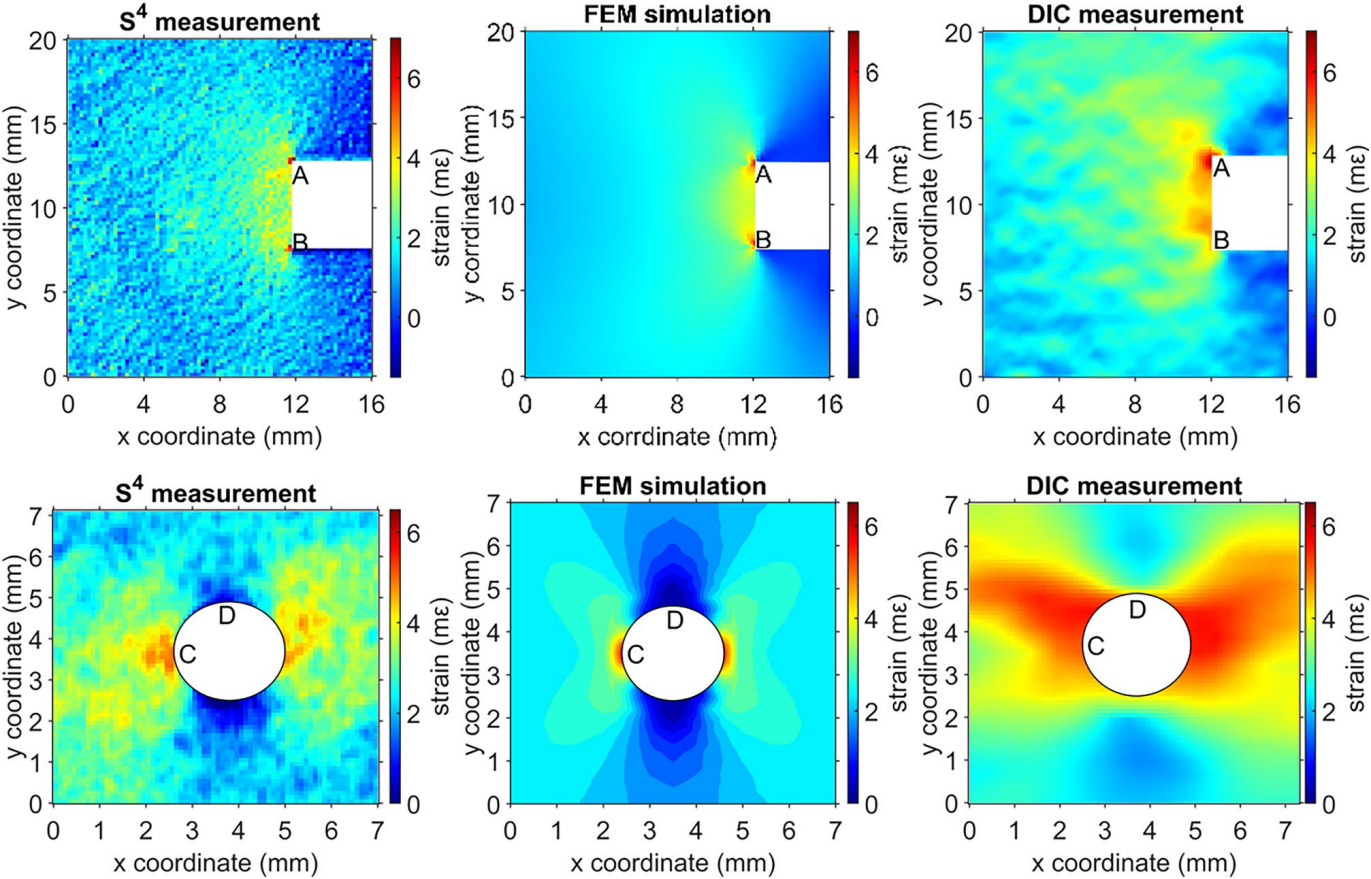
Color-coded strain maps of the acrylic specimens measured with S^4^ and DIC, and the corresponding FEM simulations, upper row: specimen with a notch on the right, lower row: specimen with a hole at the center.

For the notched specimen, the S^4^ scanned region on this specimen was a 20 × 16 mm^2^ rectangular area. To increase spatial resolution, the S^4^ read head position was scanned in steps of 0.2 mm, giving a total of 8181 points over the whole ROI. For the holed specimen, the S^4^ scanned region was a 7 × 7 mm^2^ square area with steps of 0.1 mm in each direction, giving a total of 5041 points over the ROI. We performed two scans of the specimen, one before and one after loading, and subtracted the first from the second to account for background spatial irregularities resulting from minor strains induced in the SWCNT sensors during film curing. Figure 4 shows the resulting net strain pattern as a color-coded map.

To allow direct comparison with the S^4^ results, we also measured a DIC strain map of the specimen in parallel under the same conditions. Because no out-of-plane deformation was observed in this test, we used a 2D-DIC technique with a single camera (Logitech BRIO Ultra HD). The camera had been calibrated before use in DIC measurements. For the notched specimen, the section corresponding to our ROI contained approximately 2300 × 1200 pixels. Image post-processing was performed with the Digital Image Correlation Engine (DICe), using a subset size of 31 pixels (0.4 mm) and a step size of 15 pixels (0.2 mm). Following a sensitivity study, the VSG size was selected as 120 pixels (0.15 × 0.15 mm^2^). For the holed specimen, the section corresponding to our ROI contained about 850 × 850 pixels. Image post-processing was performed using a subset of 25 pixels (0.2 mm) and a step size of 12 pixels (0.1 mm). The VSG size was determined as 90 pixels (0.08 × 0.08 mm^2^). More information on the DIC measurement can be found in Table S1.

In order to compare the experimental strain maps with a computational simulation, we built a finite element method (FEM) model using ANSYS 2021 R1. The material model assumed isotropic elastic with a Young’s modulus of 3.0 GPa, and a Poisson ratio of 0.37. To match the experimental DIC and S^4^ step size, the minimum element length near the notch and the hole was set to 0.2 mm and 0.1 mm. Figure S2 shows the FEM mesh grid used to compute the strain map.

In Fig. 4 we compare color-coded strain maps obtained by S^4^, DIC, and FEM. Both the S^4^ and DIC maps give strains with magnitudes similar to the FEM simulation result, but with significantly different spatial details. In particular, the strain map from S^4^ shows finer spatial detail and agrees more closely with the simulation results. This is evident from the two strong strain concentrations located at the inner corners of the notch (points marked A and B). S^4^ reveals highly localized strain maxima indicated by the red spots, accurately capturing the large strain gradients. These strongly resemble the localized maxima in the FEM simulation. By contrast, those maxima appear diffuse and somewhat displaced in the DIC strain map. For the specimen with the hole, S^4^ map reports much more accurate strains than DIC, particularly at the high strain gradients near the hole.

**Figure 5:**
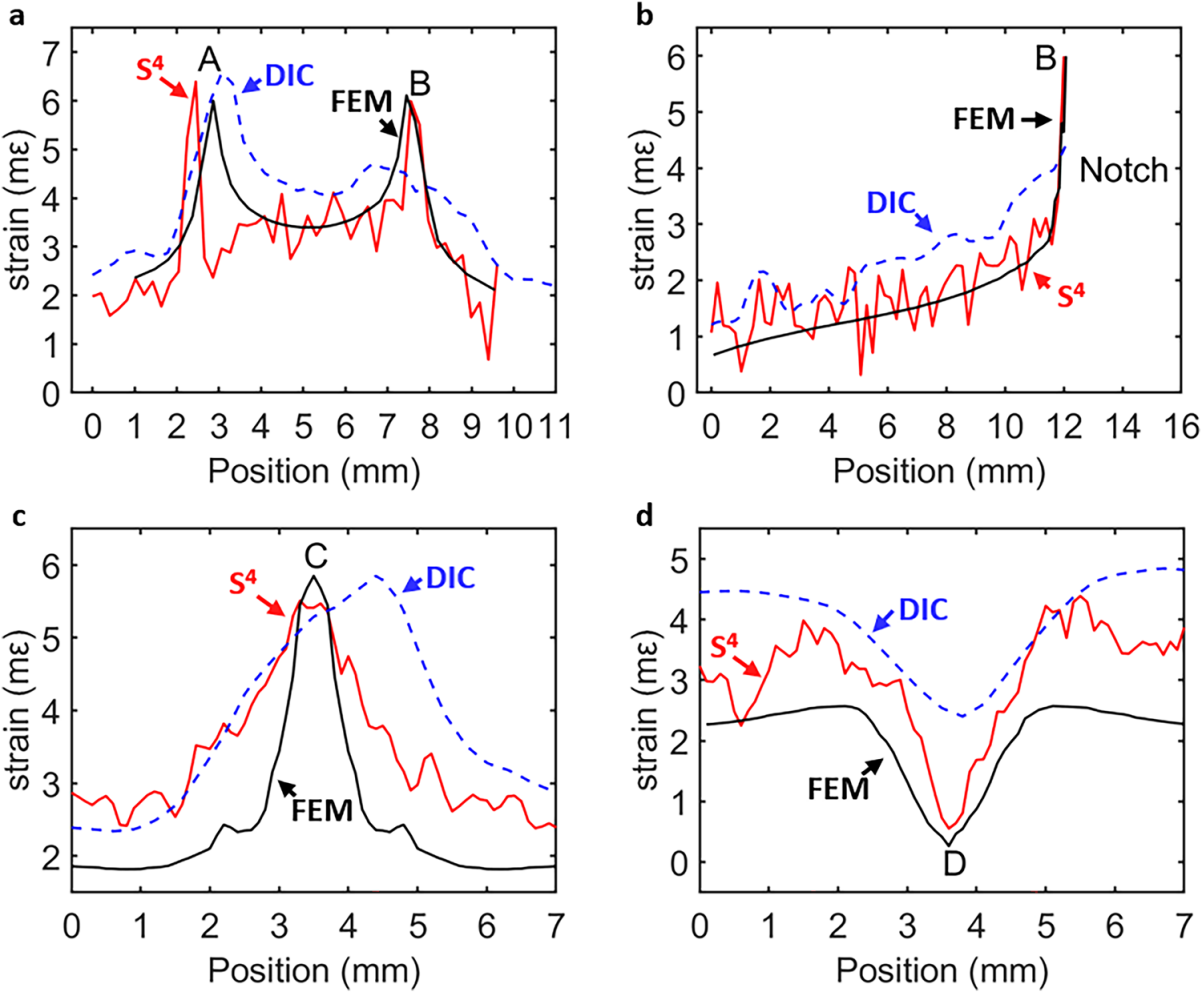
Strain profiles along (**a**) the line connecting points A and B in the top row of Fig. 4; (**b**) the horizontal line through point B in the top row of Fig. 4; (**c**) the vertical line through point C in the bottom row of Fig. 4; (**d**) the horizontal line through point D in the bottom row of Fig. 4.

Figure 5a shows strain profiles of the notched specimen along the vertical line connecting points A and B, and Fig. 5b shows strain profiles along the horizontal line passing through the bottom of the notch at point B. Comparing to the FEM profile, both S^4^ and DIC successfully capture the first peak at point A. However, the DIC peak at point B is excessively broadened, too low in magnitude, and inconsistent with the specimen’s symmetry. In Fig. 5b, DIC underestimates the peak strain at point B by nearly 2 mε.

To compare results for the holed specimen in the lower frames Figs. 4, 5c shows strain profiles along the vertical line through point C and Fig. 5d shows profiles along the horizontal line through point D. We find that S^4^ captures the magnitudes and symmetric positions of peak strain in good agreement with the FEM simulations, whereas DIC errs in position in 5c and in magnitude by nearly 2 mε in 5d. The discrepancies result from numerical errors and VSG spatial smoothing in DIC image processing.

We conclude that because it does not trade spatial resolution for strain resolution, the S^4^ method more accurately detects highly localized regions of large/peak strains (with steep strain gradients). Such strain concentrations and steep strain gradients at edges, corners and crack tips are in general not captured by DIC, yet they must be detected in structural maintenance and health inspections because they may lead to material and structural failures.

### Case 2: Concrete block

The second case study specimen was a small concrete block that had been cast with a round hole through its center to concentrate stress during compression testing. The specimen contained type I/II Portland cement with a water/cement ratio of 0.5 and a cement/aggregates ratio of 0.5 (see Fig. S3 for details). It was cured in water for 7 days before testing. We coated the central section of the specimen’s top surface with an opaque base layer to block the intrinsic near-infrared emission from cement^39^, which would otherwise interfere with S^4^ measurements. Then a DIC speckle pattern was applied, followed by an isolation layer and the S^4^ sensing layer. We also attached a conventional foil strain gauge on the side of the specimen, as shown in Fig. S3c. The specimen was then uniaxially compressed until the foil gauge gave a strain reading of 650 με.

A 20 × 20 mm^2^ region of the coated face was scanned by the S^4^ read head in steps of 0.25 mm, giving a total of 8181 points over the ROI. Emission spectra were measured at these points with laser polarization both parallel and perpendicular to the compression axis to probe strain along those directions. Following the same procedure illustrated in Fig. 1c, we performed full scans of the specimen before and after loading, and subtracted the first from the second to account for minor spatial variations in initial SWCNT strain states caused by film curing and computed strain map with − 1.5 nm/mε standard gauge factor.

DIC data on the same specimen were collected using two cameras and the 3D-DIC technique to account for possible out-of-plane motion during loading. The cameras were identical and the same type as used in the Case 1 study. Image post-processing was performed with the DICe software, using a subset size of 30 pixels (0.54 mm) and a step size of 15 pixels (0.27 mm). The virtual strain gauge size was finally selected as 120 pixels (about 0.2 × 0.2 mm^2^) after a sensitivity study.

For our FEM simulation, the material was defined as normal (Portland cement) concrete in the ANSYS Granta Design database. The material model assumed a Young’s modulus of 19.4 GPa and a Poisson ratio of 0.14. The FEM mesh grid used to compute the strain map is illustrated in Fig. S4.

**Figure 6:**
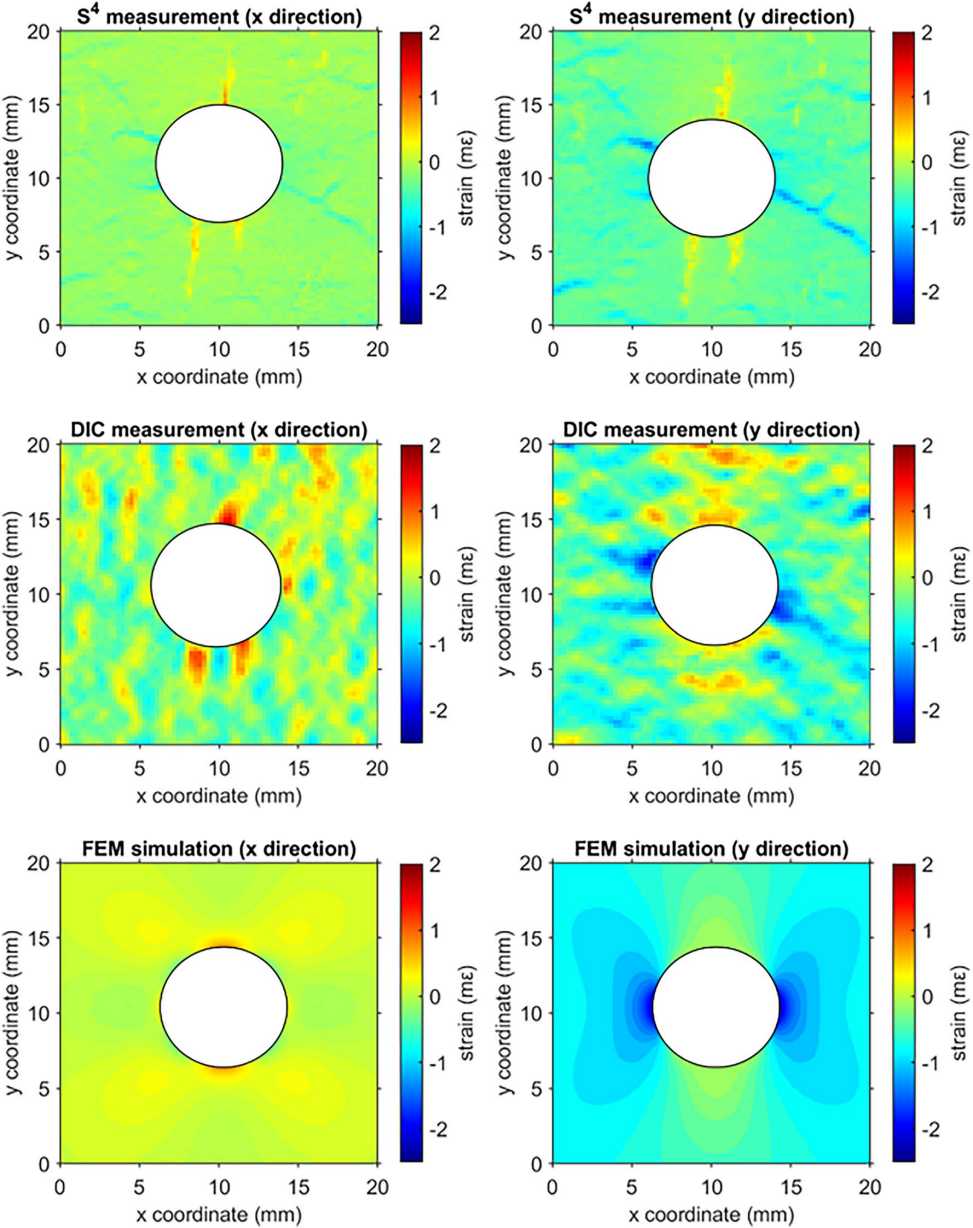
Strain maps of a concrete specimen with a central hole that was compressed along the y-axis. Rows from top to bottom show strain measured with S^4^, DIC, and simulated by FEM computations. The left panels show strain components perpendicular to the stress axis and right panels show components parallel to the stress axis.

Figure 6 compares maps of strain near the hole along the perpendicular (*x*) and parallel (*y*) axes relative to the loading direction, as measured by S^4^ and DIC methods and computed by FEM. The FEM results show that the areas on both sides of the hole along the perpendicular (*x*) axis are (1) weakly compressed (or near zero strain) at the left and right edges of the hole, and (2) in tension at the top and bottom of the hole. The FEM results near the hole along the parallel (*y*) axis clearly show (1) compression at the left and right edges of the hole, and (2) tension at the top and bottom of the hole. The tensile and compressive strain concentrations found by S^4^ and DIC measurements agree qualitatively with the FEM maps but vary significantly in detail. The S^4^ strain map is more accurate and more precise, if we examine the *x-* and *y-*axis strain maps for quantitative details and sharply defined features. However, in the DIC map many strain features are completely absent, such as the fine compression features at about 45° in the *x*-axis map. We note that because concrete is an inhomogeneous material for which fracture behavior is complex and difficult to predict, our FEM model cannot accurately capture all the features evident in S^4^ strain map. The vertical stripes in our measured S^4^ maps showing high tension strain concentrations near the hole indicate formation of microcracks during early stage material failure.

Both measurements reveal such localized strain anomalies, but DIC strain maps fail to reveal many finer details. For example, fine tension features in the vertical direction appear in the x-axis DIC map but are totally missing from the y-axis DIC map, a result inconsistent from a mechanics standpoint. By comparison, the S^4^ maps more precisely show the strain distribution in both directions that may result in the development of the microcracks, as is beneficial for fracture studies and damage detection.

### Case 3: Aluminum plates

The final case studies were performed on two 6.4 mm thick, 25.4 mm wide aluminum plates with subsurface defects. As illustrated in Fig. S5, in one, a 3.8 mm hole was drilled through the entire specimen width along the *y*-axis to create the defect; in the other, the hole extended only through one-third of the width. These studies were intended to test whether the internal structural damage represented by the holes could be detected from surface strain measurements after tensile loading along the *x*-axis. To account for possible out-of-plane motion during loading, we applied the 3D version of DIC for both specimens.

**Figure 7:**
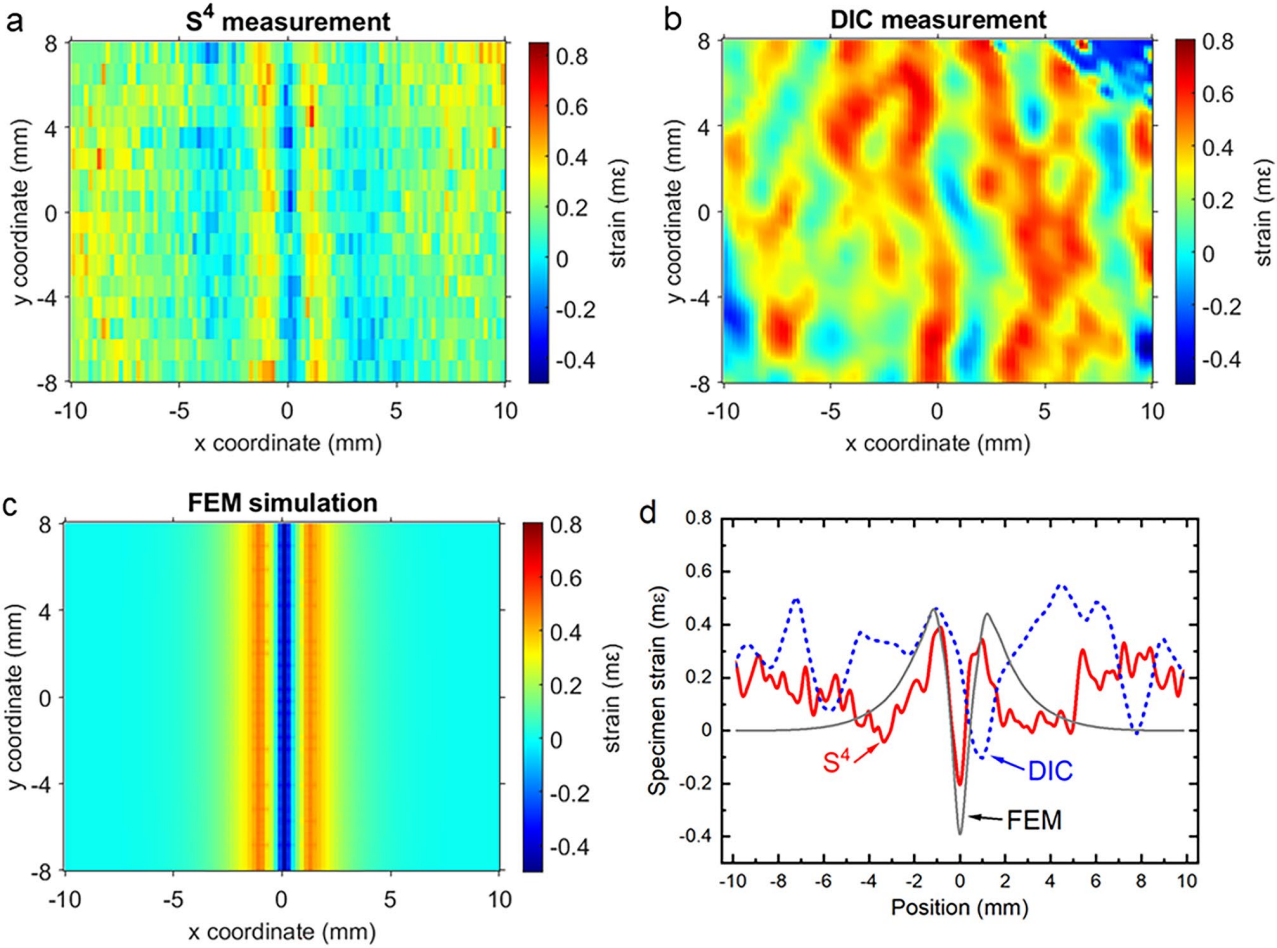
Color-coded strain maps of the subsurface drilled and stressed aluminum specimen as found from (**a**) S^4^ measurements; (**b**) DIC measurements; (**c**) FEM simulation. Frame (**d**) shows strain profiles from the three methods along the *x* direction at *y* = 0.

Figure 7 compares the results for the first specimen (with a through hole). We controlled the tensile load on the specimen to just exceed the yielding point before releasing, so that a small residual strain remained in the plate. A single band of compressive strain (blue region) sandwiched between two bands of tensile strain concentration (yellow/red regions) can clearly be identified in the S^4^ map and the FEM simulation. As shown by the strain profiles plotted in Fig. 7d, S^4^ and FEM agree well near the center, with both showing similar positive–negative–positive strain patterns, negative-going peaks at *x* = 0 mm, and amplitudes matching within 0.2 mε. Discrepancies can be seen at locations more than 5 mm from the center, where the S^4^ map suggests increased strain whereas FEM predicts strain falling to zero. The symmetric pattern in the S^4^ map suggests that this mismatch may represent a real physical effect that was not properly captured in the FEM simulation. The DIC strain map has a higher noise level than S^4^ and is much less successful in locating the main negative feature or revealing the overall strain pattern. This test therefore shows that the S^4^ method is more effective than DIC in detecting hidden damage in specimens with surface strains below 1 mε.

**Figure 8:**
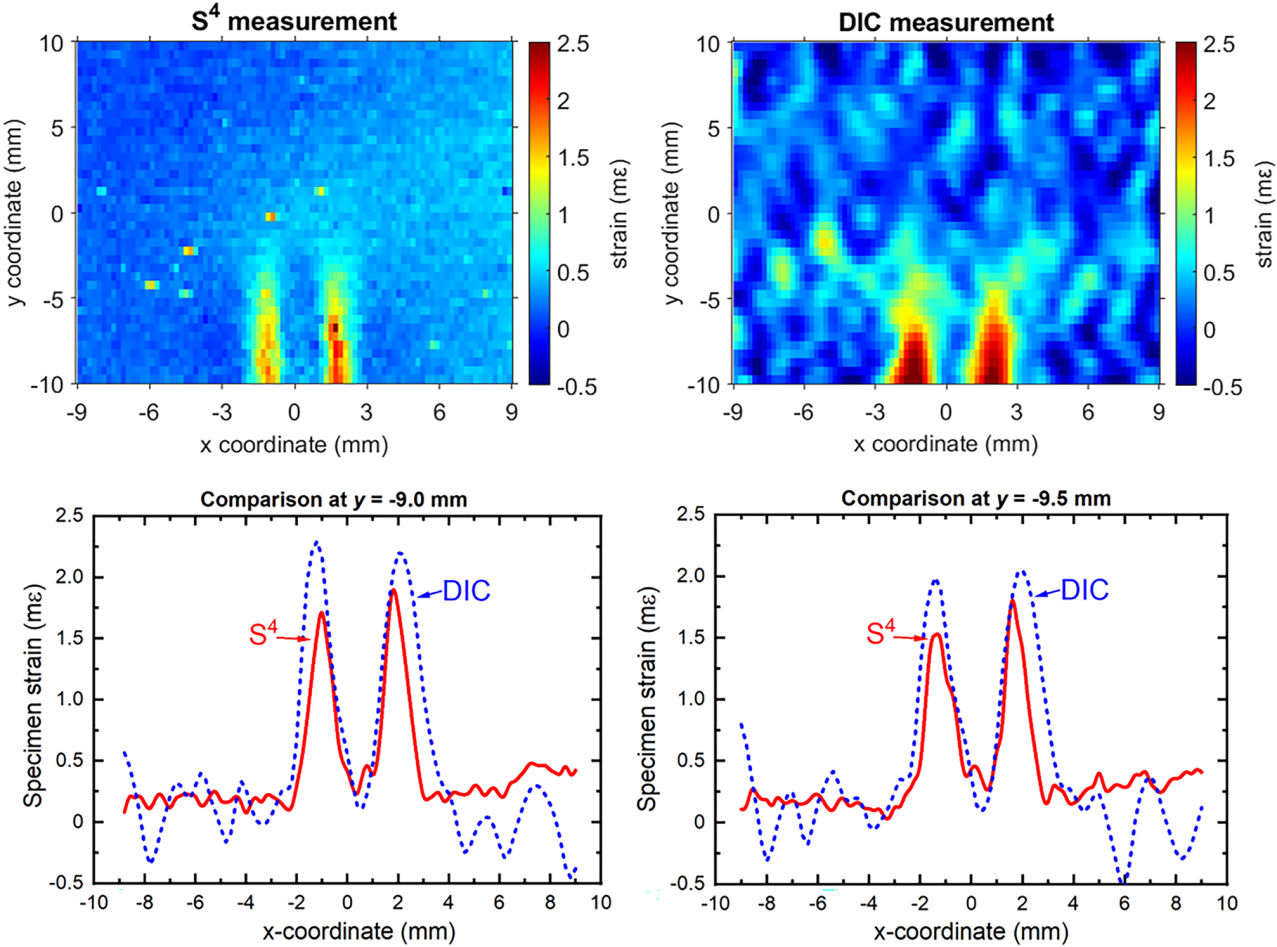
Strain maps of the aluminum specimen that had an 8 mm long hole drilled underneath the x–y surface along the *y*-direction and was then stressed in tension along the x-axis. Top left and top right frames show color-coded strain maps measured with S^4^ and DIC, respectively. Bottom left and bottom right graphs show strain profiles from the two methods along the *x* direction at *y* = − 9.0 mm and *y* = − 9.5 mm, respectively.

The final test specimen had larger strains that were induced by tensile loading after a 3.8 mm hole had been drilled through one-third of its width to represent internal (sub-surface) damage. The measurement results are shown in Fig. 8. In this case, comparison with FEM simulation was not possible because of uncertainties in the specimen’s post-yield material parameters and eccentricity in loading. The latter is evident in the S^4^ asymmetric strain map in Fig. 8. Here, there is a clear difference between right and left peak magnitudes even though they would be expected to match by symmetry. The S^4^ and the DIC strain maps agree qualitatively in showing two strips of strain concentration that are greatest at the drilled edge of the plate and gradually dissipate near the end of the drilled hole. This strain pattern reflects the subsurface structural defect. Comparing to the results shown in Fig. 7, one can see that the DIC map quality is improved for this higher strain (up to 2 mε) case. To more quantitatively display results from S^4^ and DIC, we plot strain profiles at *y* = − 9.0 mm and *y* = − 9.5 mm in the lower panels of Fig. 8. There is a considerable discrepancy of about 0.4 mε in the maximum strain values found by the two methods. The S^4^ profiles show lower noise, sharper features, and a more consistent difference between the amplitudes of the peaks at positive and negative *x*-coordinates, which we attribute to eccentric loading. The DIC profiles show compressive strain at certain locations, which is incorrect. This final test case suggests that S^4^ mapping qualitatively remains superior to DIC in this higher strain regime of up to 2 mε.

## Discussion

S^4^ strain mapping performance has been significantly enhanced by the current developments, in which the sensing film containing carbon nanotubes is separated from a blocking or primer layer by a smooth isolation layer. This design reduces initial strain variations among the nanotube sensors without the need for annealing the coating at elevated temperatures, providing a major advantage for large or heat-sensitive structures. It also permits the application of DIC speckle patterns so that S^4^ and DIC strain maps can be measured in parallel on the same specimen for validation or for a novel combined hybrid S^4^ and DIC mapping approach (currently in development). In such a hybrid approach, large DIC maps could be complemented by local S^4^ mapping of regions with steep strain gradients.

In the DIC method, strain is computed from node displacement using a shape function defined over the image elements and then further spatially averaged to reduce noise. To properly choose DIC image analysis parameters, a sensitivity study is required involving readings from a reference strain gauge. By contrast, the S^4^ method measures strain directly from the spectral response of many independent sub-micrometer carbon nanotube strain sensors. The standard gauge factor of − 1.5 nm/mε is used to compute the strain map. Reference gauges are not needed, and there is no trade-off between strain resolution and spatial resolution.

Additional important point is that DIC does not provide the strain values at edges; the values in our DICe strain maps are points close to but not exactly at the edges (excluding edge VSG due to missing speckles close to the edges).

A great advantage of S^4^ is that each local strain value is independently determined and does not depend on nearby measurements. So S^4^ is able to properly measure strain values at edges with steep strain gradients.

## Conclusions

We have developed a next-generation multilayer film for non-contact spectroscopic strain mapping using the S^4^ method. In this three-layer design, the substrate is coated first with an opaque primer layer, then with a smooth polymeric isolation layer, and finally with a thin film containing single-wall carbon nanotube strain sensors. A clear topcoat layer may also be added if needed for environmental protection. Using these next-generation S^4^ films, we performed both scanned S^4^ and DIC measurements in parallel on acrylic, concrete, and aluminum test specimens that were shaped and stressed to induce systematic patterns of residual strain. Comparisons among the S^4^ maps, DIC maps, and FEM simulations show that strain patterns are more faithfully revealed by S^4^ than DIC maps, particularly for large or peak strains with steep gradients. More importantly, S^4^ is a direct method that measures strain independently at each measured point, whereas DIC is an indirect method that relies on high resolution images and image processing wherein the strain is computed by averaging over a region representing a virtual strain gauge. Such filtering can obscure important features with high strain gradients.

In addition to these benefits, S^4^ has the advantage compared to DIC of measuring accumulated strain without the need for constant observation or highly precise registration of pre- and post-stress specimen images. This feature makes it well suited to many field applications for which DIC may be impractical. Taken together, our findings point to the value of S^4^ strain measurements as a very promising alternative or complement to existing technologies for non-destructive evaluation and structural health maintenance^40^.

## Supplementary Information

Supplementary Information.

## Data Availability

Data described in this paper are available on request.
