# Supplementary material for: Next-generation 2D optical strain mapping with strain-sensing smart skin compared to digital image correlation
Source: Sci Rep. 2022 Jul 3;12:11226. doi: 10.1038/s41598-022-15332-1 (PMC9250928; doi:10.1038/s41598-022-15332-1)
Supplement: Supplementary file 1 — Supplementary Information. [file 41598_2022_15332_MOESM1_ESM.docx]

**Supplementary Information**

**Next-Generation 2D Optical Strain Mapping with
Strain-Sensing Smart Skin Compared to Digital Image Correlation**

Wei Meng ^a^, Ashish Pal ^a^, Sergei M. Bachilo ^b^, R. Bruce Weisman ^b,c^, and Satish Nagarajaiah ^a,c,d*^

^a^ Department of Civil and Environmental Engineering, Rice University, Houston, Texas, 77005 USA

^b^ Department of Chemistry, Rice University, Houston, Texas, 77005 USA

^c^ Department of Materials Science and NanoEngineering, Rice University, Houston, Texas, 77005 USA

^d^ Department of Mechanical Engineering, Rice University, Houston, Texas, 77005 USA


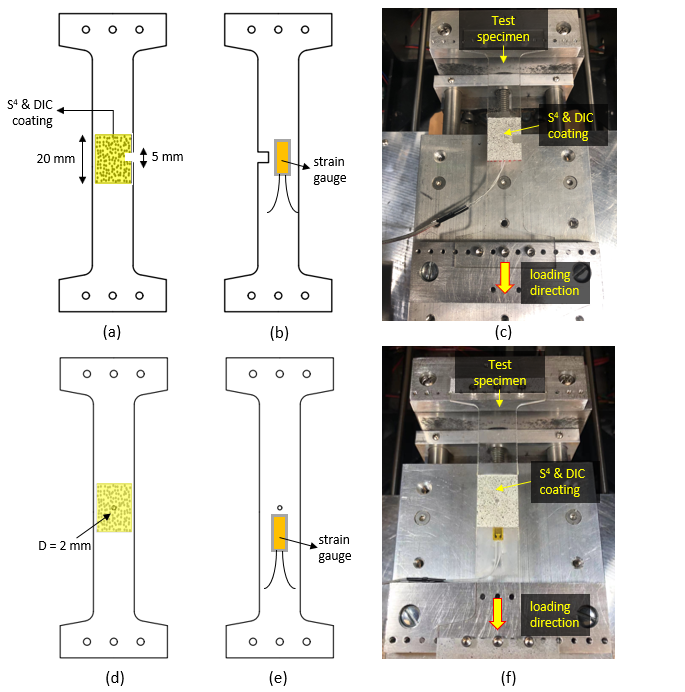


**Figure S1.** (a) Front view of the acrylic test specimen with a notch showing location of the S^4^ coating, DIC speckle pattern, and notch; (b) back view of the notched acrylic specimen showing location of the foil strain gauge; (c) photo of the notched specimen mounted on the loading jig; (d) front view of the holed specimen showing the location of the S^4^ coating; (e) back view of the holed specimen showing the location of the foil strain gauge; (f) photo of the holed specimen mounted on the loading jig.

**Table S1.** DIC Camera Details

| **Resolution** | **Chroma** | **Sensor type** | **Sensor size** |
| --- | --- | --- | --- |
| 3840 × 2160 | Color | CMOS | 4.8 mm × 3.6 mm |
| **Focal length** | **Field of view** | **DIC shape function** | **DIC gray level interpolation function** |
| 5 mm | 45º | First order | Cubic convolution |


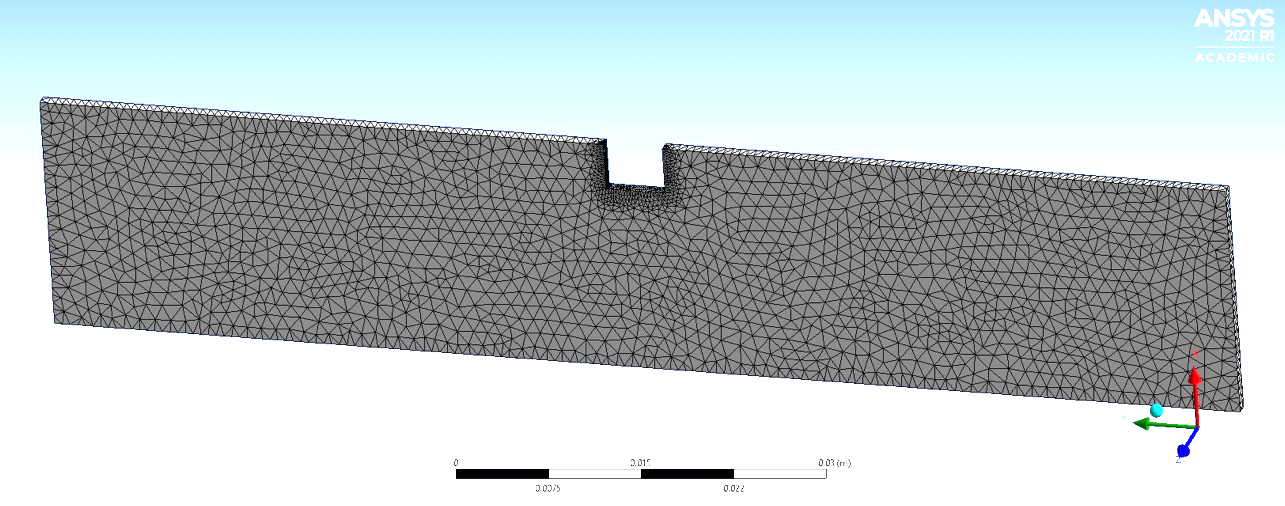


(a)


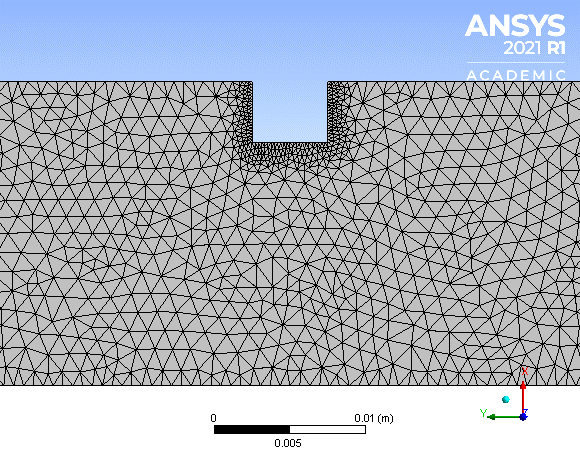


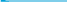


(b)


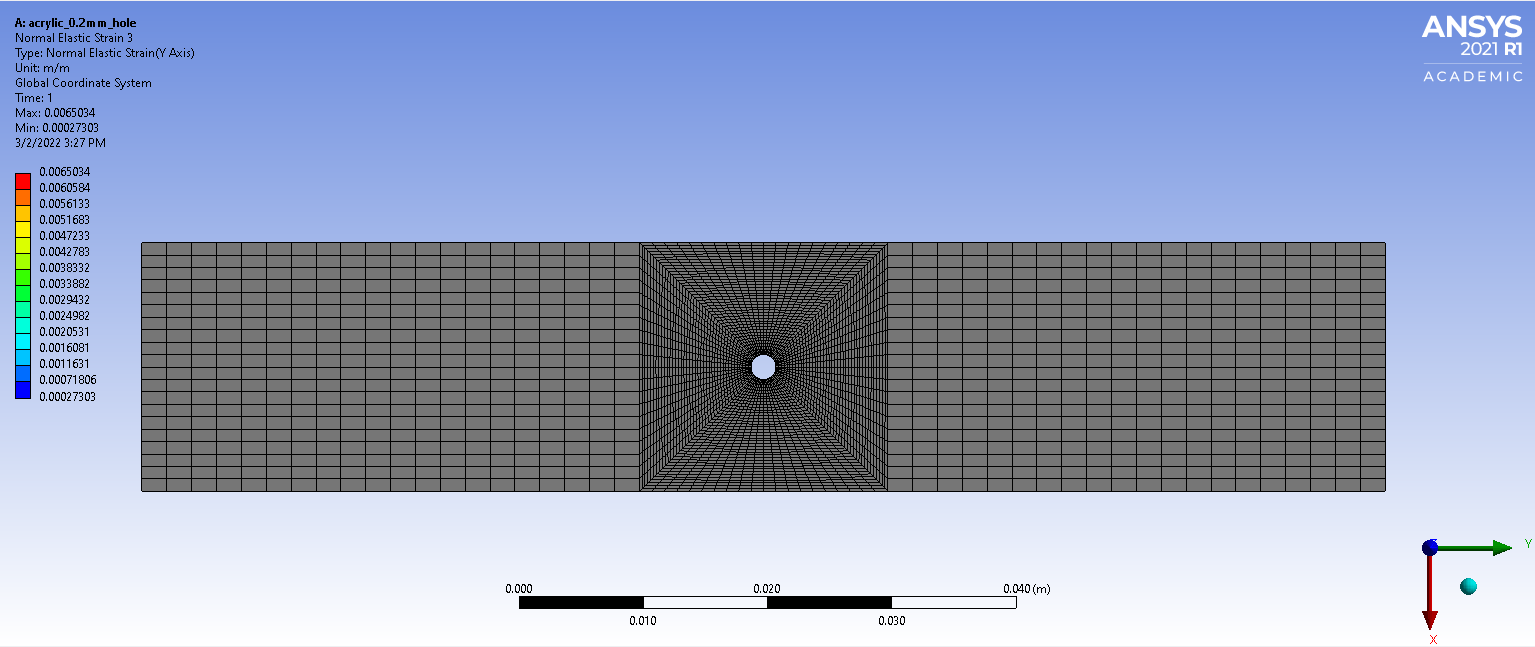


(c)


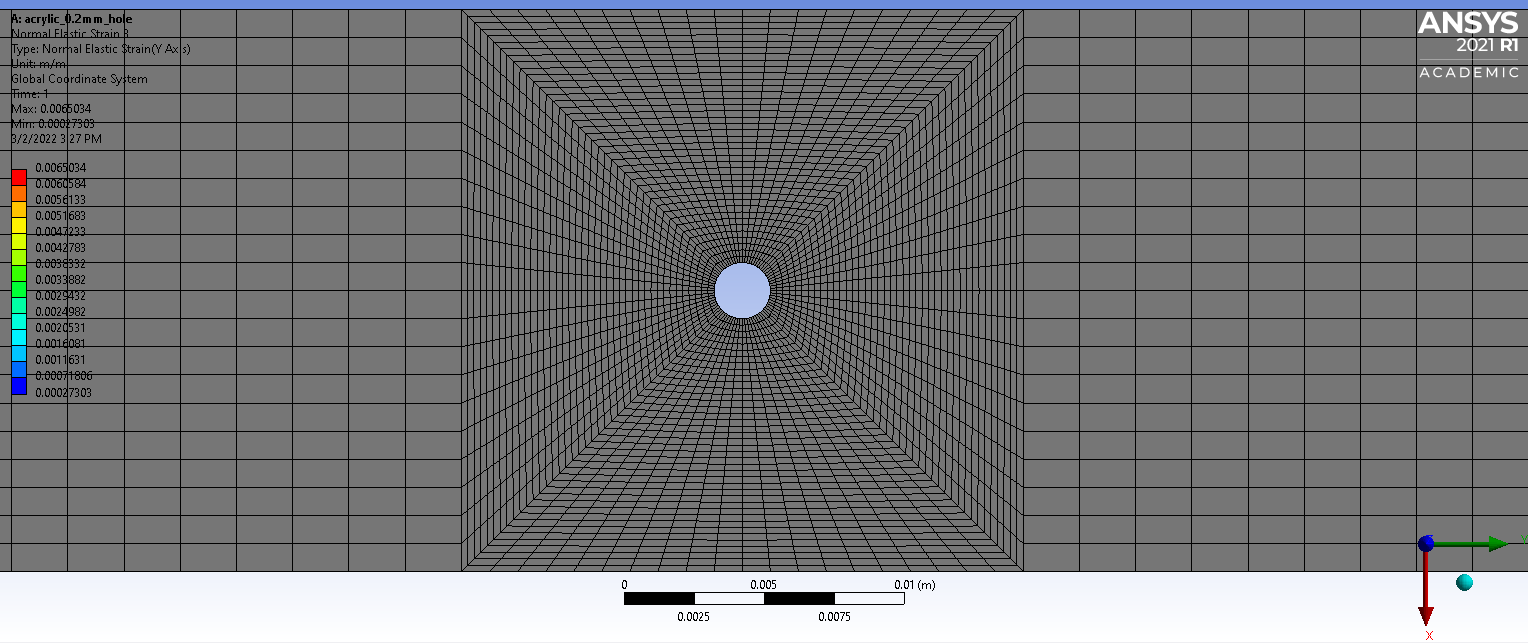


(d)

**Figure S2.** The FEM mesh grid used to compute simulated strain maps. (a) global mesh grid of the notched specimen; (b) refined mesh grid near the specimen’s notch; (c) global mesh grid of the holed specimen; (d) refined mesh grid near the specimen’s hole.


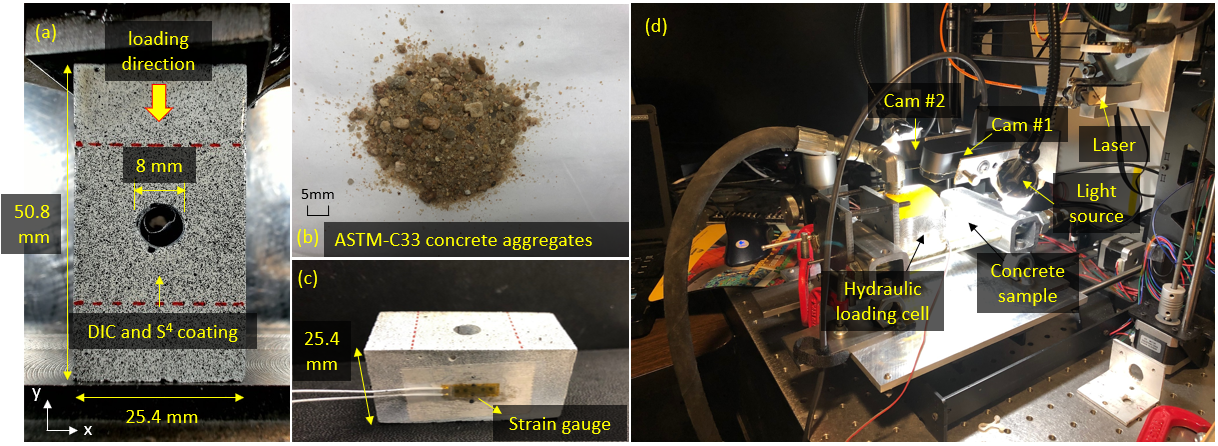


**Figure S3.** (a) Concrete specimen with an 8 mm diameter through-hole and S^4^ and DIC coatings on its front face; (b) aggregates of various sizes that were used for casting the concrete specimen; (c) concrete specimen with a foil strain gauge attached to its side; (d) experimental setup for specimen loading and data acquisition. Cam #1 and Cam #2 are the cameras used for DIC imaging.


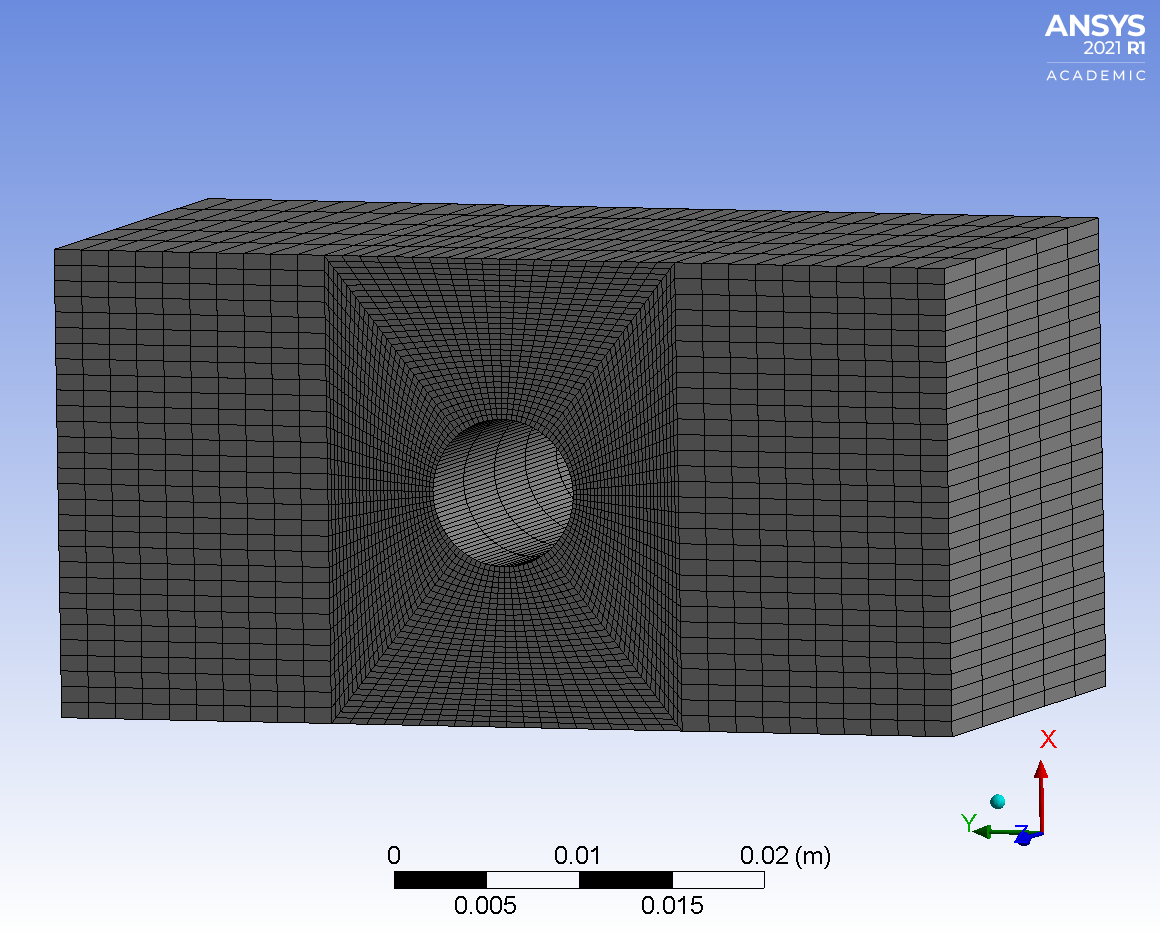


**Figure S4**. The FEM mesh grid used to compute simulated strain maps for the concrete test specimen shown in Figure S3.


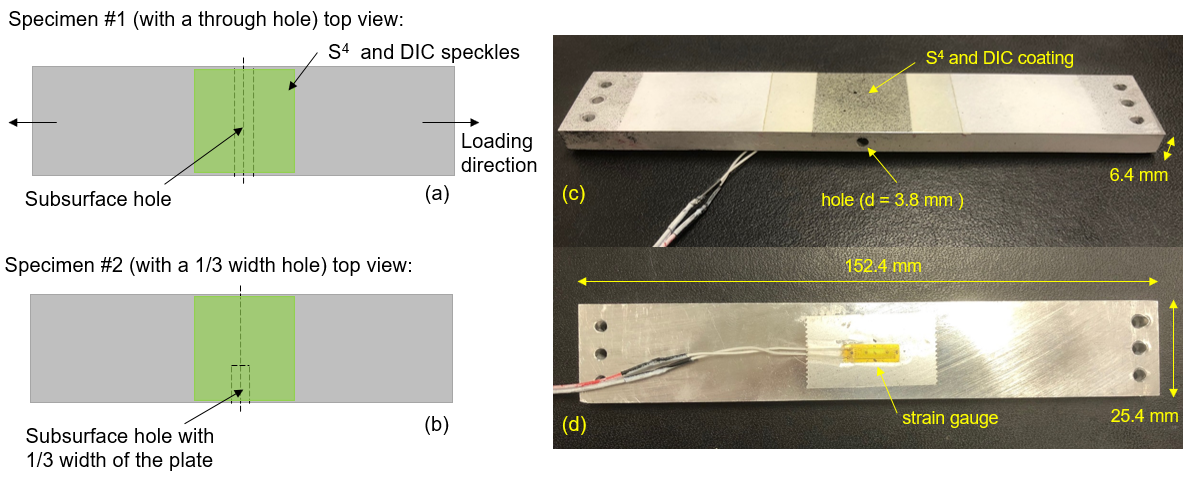


**Figure S5**. (a) Sketch of the aluminum plate specimen with a 3.8 mm sub-surface through-hole; (b) Sketch of the specimen with a sub-surface hole drilled through one-third of its width; (c) Front view of the specimen showing locations of the S^4^ coating, DIC speckle pattern, and sub-surface hole; (d) back view of the aluminum specimen showing location of the foil strain gauge.
